# Regulation of Mdm2 mRNA Stability by RNA-binding Protein RNPC1

**DOI:** 10.18632/oncotarget.1185

**Published:** 2013-07-15

**Authors:** Jin Zhang, Enshun Xu, Xinbin Chen

**Affiliations:** Comparative Oncology Laboratory, University of California at Davis, Davis, CA; Comparative Oncology Laboratory, University of California at Davis, Davis, CA; Comparative Oncology Laboratory, University of California at Davis, Davis, CA

**Figure 1:**
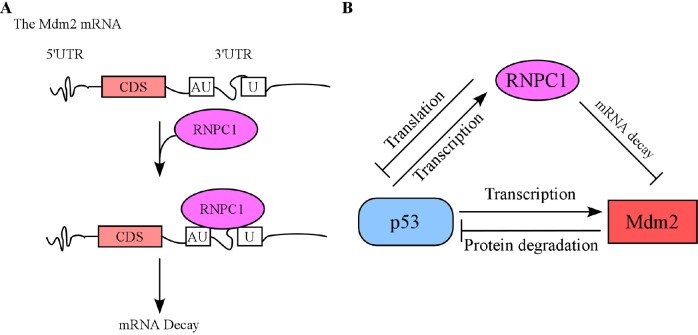
(A) A proposed model for RNPC1-mediated Mdm2 repression. (B) A proposed model for the interplay among RNPC1, Mdm2, and p53.

The murine double minute-2 (Mdm2) oncoprotein is an E3 ligase and a key regulator of a variety of fundamental cellular processes [1]. Mdm2 was originally identified as being amplified on double-minute chromosomes in transformed mouse fibroblasts [2]. Soon after its discovery, Mdm2 was found to be a negative regulator of tumor suppressor p53. The importance of Mdm2 in controlling the p53 activity is demonstrated in Mdm2 knockout mice. Mdm2-null embryos die very early during gestation, but additional deletion of p53 rescues them from death. Nevertheless, Mdm2 has p53-independent activities in promoting tumorigenesis. Due to its critical role in tumor development, understanding the mechanism by which Mdm2 expression is controlled will lay the foundation for therapeutic strategies by targeting Mdm2 for cancer management. The RNPC1 gene, also called RBM38, encodes an RNA-binding protein and is expressed as two isoforms, RNPC1a and RNPC1b. Both RNPC1a and RNPC1b contain a putative RNA recognition motif, which shares a high homology with the ones in HuR and Musashi. Recently, we showed that RNPC1 is a target of p53 and can in turn translationally repress p53 expression [3,4]. Thus, RNPC1 forms a feedback-regulatory loop with p53. In a recent publication, we described a novel regulation of Mdm2 by RNPC1 via mRNA stability [5] (Fig. 1A). In particular, we generated several stable cell lines that can inducibly express ectopic RNPC1 under the control of tetracycline-responsive promoter. We found that upon induction, ectopic RNPC1 is able to inhibit Mdm2 expression at the level of transcript and protein regardless of p53. Consistent with this, knockdown or knockout of endogenous RNPC1 led to an increased level of Mdm2 transcript and protein independent of p53. Mechanistically, we found that RNPC1 is able to bind multiple AU-/U-rich elements in Mdm2 3'untranslated region (3'UTR) and subsequently, destabilizes Mdm2 transcripts. We also found that the AU-/U-rich elements in Mdm2 3'UTR and the RNA-binding domain in RNPC1 are required for RNPC1 to inhibit Mdm2 expression. Together, these data suggest that RNPC1 is a critical regulator of Mdm2 and further studies are needed to address that the biological significance of these regulation under a physiological setting. The finding that RNPC1 destabilizes Mdm2 transcripts may add an additional level of control to p53-Mdm2 autoregulatory feedback loop, which dictates cellular p53 levels and activity (Fig. 1B). Briefly, RNPC1 is a target of the p53 and a negative regulator of p53 and Mdm2. Thus, it is likely that under non-stress conditions, RNPC1 and Mdm2 restrain p53 expression via translational repression and protein degradation, respectively. In response to various stimuli such as DNA damage, p53 is accumulated and transactivates both RNPC1 and Mdm2. Consequently, RNPC1 induction leads to decreased Mdm2 expression, which would release p53 from Mdm2-mediated inhibition. We would like to mention that although RNPC1 inhibits p53 translation, this regulation may be less effective under stress conditions owing to a global inhibition of protein synthesis. In addition to RNPC1, Mdm2 can be regulated by several other RNA-binding proteins at posttranscriptional level. For example, Mdm2 is regulated by HuR via mRNA stability [6]. In addition, Mdm2 is found be regulated by La protein via protein translation, which may play a critical role in leukemogenesis [7]. Furthermore, a number of ribosomal proteins, such as RPL5, RPL7, RPL11, RPL23, and RPS27L, are found to interact with Mdm2 and inhibit its E3 ligase activity [1]. Since altered expression of RBPs has been found in many types of human cancer, understanding how Mdm2 is regulated by RBPs and whether this regulation contributes to tumorigenesis might provide novel therapeutic strategies for cancer treatment. In summary, our data suggest that RNPC1 is a critical regulator of Mdm2. As both RNPC1 and Mdm2 expression is altered in several types of human cancers, this regulation may represent an important mechanism for cancer development and RNPC1 may be used for targeting tumors with high Mdm2 expression.
